# Conformational changes in amyloid-beta (12–28) alloforms studied using action-FRET, IMS and molecular dynamics simulations†
†Electronic supplementary information (ESI) available: The structure of the donor and acceptor chromophores, representative structures and Ramachandran plots for the N-terminal donor doubly-grafted species complimentary to Fig. 2 and 3, and numerical values of the CCS and action-FRET data in Fig. 1. See DOI: 10.1039/c5sc01463h


**DOI:** 10.1039/c5sc01463h

**Published:** 2015-06-18

**Authors:** Steven Daly, Alexander Kulesza, Frederic Poussigue, Anne-Laure Simon, Chang Min Choi, Geoffrey Knight, Fabien Chirot, Luke MacAleese, Rodolphe Antoine, Philippe Dugourd

**Affiliations:** a Université de Lyon , F-69622 , Lyon , France; b CNRS et Université Lyon 1 , UMR5306 , Institut Lumière Matière , France . Email: philippe.dugourd@univ-lyon1.fr; c CNRS et Université Lyon 1 , UMR 5280 , Institut des Sciences Analytiques , France

## Abstract

Small oligomers of the amyloid beta protein (Aβ) have been implicated as the neurotoxic agent leading to Alzheimer's disease, and in particular mutations in the hydrophobic core region comprising amino acids L17 to A21 have a large influence on the propensity for aggregate formation. It has been shown that the F19P alloform of Aβ forms small aggregates, but does not proceed to form large fibrils and plaques. In order to understand the origin of this behavior, the gas phase conformations for the different charge states of the wild-type 12–28 fragment of the amyloid beta and its F19P alloform were studied by a combination of action-FRET, ion-mobility spectrometry (IMS) and molecular dynamics simulations. Comparison of the experimental and theoretical action-FRET efficiencies and collision cross sections allowed the determination of the lowest energy conformational family for each alloform and charge state. For both alloforms, it was found that there is a change from globular to helical structure between the 3+ and 4+ charge states. Additional protonation to give 5+ and 6+ charge states caused unfolding of this helical motif, with the wild alloform showing β-turn like motifs and the F19P alloform random coil motifs. The presence of the helical to β-turn structural transition in the wild, but not the F19P, alloform may help to elucidate the origin of the large difference in aggregation behavior of the two alloforms.

## Abstract 2

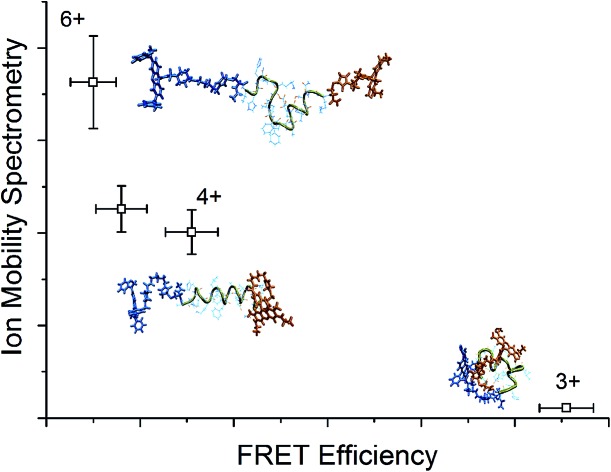
The gas phase conformations of two amyloid beta mutants are studied by multiple techniques to elucidate the origin of the different aggregation behaviour.

## 1 Introduction

The amyloid β-protein (Aβ) is strongly implicated as the main neuropathic agent in Alzheimer's disease; it has been known for over three decades that plaques formed of Aβ are found in the brain tissue of patients suffering from the disease.^1^ The observation of pervasive plaques led to the suggestion that they were the causative agent of the disease. However, recent developments have shown that small soluble Aβ oligomers retain their neurotoxicity even in the absence of large aggregates and plaques.^2^,^3^ Even more surprising, it was found that it was not necessary to have the full 40–42 amino acid protein in order to form aggregates or to observe neurotoxic effects, with many protein fragments also exhibiting these features.^4^,^5^ In particular, the region known as the “hydrophobic core” – from L17 to A21 – is found to be essential for the formation of large aggregates and fibrils in the full Aβ protein.^6^ It is also found that mutations in or close to the hydrophobic core region have a large influence on the propensity for aggregate formation.^7^–^10^ An example of this is the mutation Phe19 → Pro19, which prevents the formation of fibrils.^11^,^12^ Indeed, Wood *et al.* found that the substitution of any of the residues within the hydrophobic core with proline completely inhibits the formation of fibrils.^11^

The 12–28 fragment of the Aβ protein (Aβ_12–28_, VHH QKLVF FAEDV GSNK) has attracted attention since it exhibits essentially identical neurotoxic behavior and fibril formation as the full Aβ protein.^13^ As such, the conformational characteristics have been studied extensively. Graslund *et al.* found *via* NMR and CD measurements that Aβ_12–28_ shows an extended polyproline II (PII) conformation at low temperature and a random coil structure at high temperature.^14^,^15^ Several molecular dynamics studies have been performed, which have also shown there exists a transition from extended to folded structure as a function of the solvent polarity and temperature.^16^–^18^ There is also a strong pH dependence of the structure and nature of aggregation, in particular a strong dependence between the formation of random coil or helical motifs, and an equally strong dependence on the aggregation properties, which are found to preferentially form between pH 4–7, and thus when a high protonation state is favoured.^19^–^21^ There also exists a gas phase study of Aβ_12–28_ using a combination of IR spectroscopy, ECD and IMS to elucidate the difference between different protonation states, with a transition from compact to extended forms seen when adding a proton from 2+ to 3+ charge states.^22^

The gas phase provides an ideal setting in which to study the conformational changes brought about by the protonation state of a peptide or protein due to the ability to effectively isolate and study a single charge state. It is now well established that is possible to preserve intact molecular edifices in the gas phase through soft ionization techniques such as electrospray ionization, and indeed there exist several gas phase studies in the properties of Aβ monomers and their aggregates using ion-mobility mass-spectroscopy (IMS).^12^,^22^–^27^ IMS gives access to information on the global structure and is particularly sensitive to changes in the overall structural motif of a system. A complimentary technique that has been recently developed – action-FRET – provides information on the separation of two specific regions of a protein by measurement of the FRET efficiency as reported by specific photo-fragmentation of the acceptor chromophore following specific excitation of a donor chromophore.^28^ Although action-FRET is sensitive to global changes in structural motifs, it is also able to detect much smaller changes within the same structural motif due to the 1/*R*^6^ dependence of FRET efficiency on chromophore separation. In addition to these experimental techniques, molecular dynamics (MD) simulations of biomolecules have been shown to produce a biologically relevant picture of folding and aggregation in amyloid-β.^29^ The combination of the three techniques thus has the potential to glean additional information that either technique alone is unable to access.

In this paper, results of IMS and action-FRET measurements on two alloforms of Aβ_12–28_ cations in different protonation states are presented; wild-type (WT) Aβ_12–28_ and its F19P alloform. Comparison of FRET efficiency and collision cross section (CCS) for the charge states of these two alloforms with theoretical models allows the identification of the dominant structural families for the charge states of each alloform. These structural families are compared for the two alloforms with the view of gaining insight on their different aggregation behavior based upon the changes in structural motifs with protonation state the monomer.

## 2 Method

### 2.1 Peptides and chromophores

Carboxyrhodamine 575 C_5_-maleimide (Setareh Biotech) (R575) and QSY 7 C_5_-maleimide (Life Technologies) (QSY7) – the structures of which are shown in Fig. S1† – were used as donor and acceptor chromophores for action-FRET measurements respectively. Both have been previously characterized in the gas phase; R575 absorbing at 505 nm and QSY7 absorbing at 545 nm.^28^,^30^ The chromophores are designed to be grafted onto thiols, and thus the target peptide must contain 2 cysteine residues.

WT Aβ_12–28_ with capped N- and C-terminal cysteine residues (Ace-CV^12^HHQKLVF^19^FAEDVGSNK^28^C-NH_2_) and the F19P alloform (Ace-CV^12^HHQKLVP^19^FAEDVGSNK^28^C-NH_2_) were purchased from GeneCust (Luxembourg) and each dissolved in a 1 : 1 mixture of H_2_O : acetonitrile to a concentration of ∼500 μM. Stock solutions of each dye were made by dissolving to concentrations of ∼10 mM concentration in DMSO and 10 μl of each these solutions were added to the peptide solution. This solution was left at room temperature for one hour to allow the reaction to run until completion. For use in the electrospray source, the reaction solutions were further diluted to concentrations of ∼50 μM. In order to produce the 6+ charge state, 0.1% acetic acid was added to the electrospray solution.

### 2.2 Mass spectrometry and optical spectroscopy

A linear quadrupole ion trap mass spectrometer (LTQ Velos, Thermo Fisher Scientific, San Jose, CA) was used to generate, mass select and trap ions in a first, high pressure ion trap for a controlled duration. During ion trapping, ions can be activated and fragmented by collisions (CID) or photons (LID). Fragment ions are transmitted to a second ion trap, with a low pressure, where they are mass analyzed. A fused silica window (3 mm thick, 1 inch diameter) is positioned at the back end of the instrument and allows for the introduction of the lasers in the UV-Visible range along the ion traps axis. 1–2 mm diameter circular openings in trapping-electrodes enable the interaction of the trapped ions with an on axis laser beam. In order to optimize laser transmission, the central hole of the electrode closest to the fused silica window was enlarged to 5 mm in diameter.

The light source used is a Panther EX OPO pumped by the third harmonic (355 nm) of a Surelite II Nd:YAG laser (Continuum, Santa Clara, CA). A repetition rate of 10 Hz and pulse-widths of the order of 5 ns were used. The visible portion of the spectrum was used directly *via* the signal beam of the OPO (410–700 nm), which is collimated and refocused with a long focal distance lens of 500 mm. Pulse energies were kept between 1.0 to 4.5 mJ per pulse to avoid saturation. A mechanical shutter, synchronized with the mass spectrometer, is used to stop the beam at all times except the ‘ion activation window’ – that is the time after ion accumulation and before the mass analysis. A single laser pulse was used for the irradiation of the trapped ions. When irradiating ions the normalized collision energy is kept at zero.

For action-FRET measurements, mass spectra were accumulated for 2 minutes, and the measurement repeated 5 times to give a total irradiation time of 10 minutes for each charge state and wavelength. Measurements were taken at wavelengths of 505 nm and 545 nm, corresponding to the absorption maxima of the donor and acceptor chromophores respectively. The position of the beam was monitored throughout the measurements using the Guidestar II auto-alignment system. This system uses two 90 : 10 beam splitters to direct a fraction of the beam onto two CCD cameras, allowing real-time monitoring of the beam position, and to ensure the alignment could be easily reproduced for the two different wavelengths. The power was measured immediately before and after each measurement using a power meter (Ophir-Spiricon GmbH, Ahrensburg, Germany) for the duration of 1 minute and an average value for the power during the measurement determined by taking the average of the power measured immediately before and immediately after accumulation of a mass spectrum.

Action-FRET measurements were performed using the methodology described previously.^28^ This technique relies upon the measurement of the relative intensities of photo-fragments that are specific to the acceptor chromophore at wavelengths of 545 nm and 505 nm, corresponding to the absorption maximum of QSY7 and R575 respectively. The fragmentation at 545 nm is used as a reference. As QSY7 absorbs only weakly at 505 nm, the intensity of acceptor specific photo-fragments at this wavelength can be correlated to the efficiency of the energy transfer between the two chromophores, and thus to their separation. To extract the action-FRET efficiencies, the fragmentation yield was normalized to the photon flux for each measurement. The action-FRET efficiency is then calculated as ratio of the averaged fragmentation yield at 505 nm to the averaged fragmentation yield at 545 nm. Finally, to account for the influence of direct absorption and fragmentation of the acceptor at 505 nm a correction of – 0.25 (corresponding to the ratio of the fragmentation efficiency of the acceptor chromophore at 505 and 545 nm) was applied to all FRET efficiencies.

### 2.3 Ion mobility mass spectrometry

Ion mobility (IMS) measurements were performed using a custom-built ion mobility spectrometer already described elsewhere.^31^,^32^ Briefly, a 1 m long drift tube is inserted between an electrospray ionization source and a time-of-flight (ToF) mass spectrometer. Helium at a pressure of 15 Torr is maintained in the drift tube, and the temperature of the whole setup is kept at 300 K. Ions are periodically injected in the drift tube from an hourglass funnel ion trap. Their mass-to-charge ratio and drift time through the tube are simultaneously measured using the ToF. Ion mobilities and collision cross section (CCS) are finally calculated from the evolution of the ion arrival time distribution as a function of the inverse drift voltage.

### 2.4 Computational

Replica-Exchange Molecular Dynamics (REMD) was performed based on the AMBER99 force field completed with the generalized Amber Force Field (GAFF) to describe the non-standard chromophore grafted cysteine residues.^33^–^35^ Structural sampling was started from extended conformations of chromophore-grafted WT and F19P mutant sequences. Replica-exchange molecular dynamics as implemented in Gromacs 4.6.5 was used for this task using the Velocity-Verlet algorithm with a time-step of 1 fs and without any cut-offs for the evaluation of interactions.^36^–^38^ The simulation was conducted with 20 trajectories in parallel which were assigned the temperatures 220, 236, 254, 272, 292, 314, 337, 362, 389, 417, 448, 481, 517, 555, 596, 640, 687, 737, 792 and 850 K in frame of the velocity-rescaling algorithm.^39^ Exchange attempts between adjacent replicas were performed every 200 steps. This temperature range ensured suitable exchange probabilities throughout the REMD. After local optimization and a short 100 ps equilibration at each temperature, a 10 ns REMD was started and every 1000th structure of the 220 K and 292 K ensembles were taken as samples.

The lowest-energy optimized structure of the 220 K ensemble was taken as new starting point and the procedure was repeated until the potential energy distribution and the energy of the optimized lowest-energy structure had converged. The calculation of FRET-efficiency exploited Förster theory as detailed in references.^40^,^41^ The distance distribution between the optically active units of the chromophores is calculated by evaluation of the geometrical centre of all atoms in the xanthene moiety and the N-linked side chains. The FRET-efficiency distribution is then calculated from the chromophore distance and orientation distributions, and the ensemble-averaged value determined for comparison with the experimental data. The CCS for the comparison with ion-mobility data were obtained by invoking the exact hard-spheres scattering method originally proposed by Shvartsburg *et al.* and is implemented locally.^42^

## 3 Results and discussion

Aβ_12–28_ contains four basic amino acids; H13, H14, K16 and K28, and in addition both chromophores possess a single positive charge. Four different charge states are observed after electro-spraying solutions containing doubly grafted Aβ; 3+, 4+, 5+, and 6+ corresponding to there being 1, 2, 3, or 4 protons on the peptide respectively, and with the 4+ and 5+ charge states being the most abundant species. This is consistent with previous gas phase measurements of Aβ_12–28_ in which predominantly 2+ and 3+ species were observed.^22^ This indicates that the presence of the charged chromophores is not greatly perturbing the charge state distribution of the un-grafted Aβ_12–28_ peptide.

**Fig. 1:**
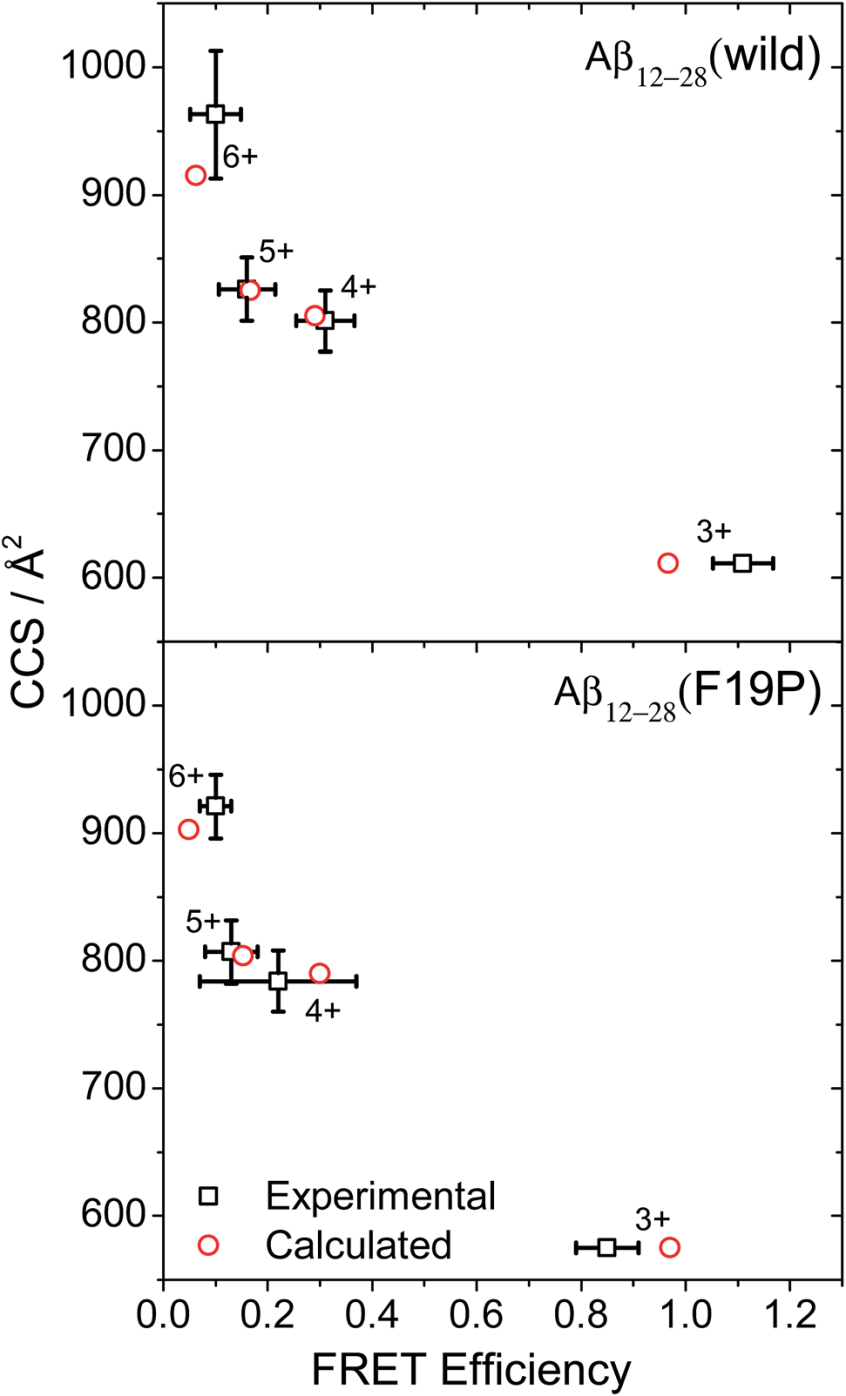
CCS *versus* FRET-efficiency for the 4 charge states of the WT (top) and F19P (bottom) alloforms of Aβ_12–28_. The black squares show the experimental data points, whilst the red circles show the average values of the conformational ensemble calculated by REMD.

The CCS and FRET efficiency for each charge state of the two doubly grafted Aβ_12–28_ alloforms were determined *via* IMS and action-FRET measurements respectively and are shown as the black squares in Fig. 1. Here, the cross section is plotted as a function of the FRET efficiency for each charge state of the wild-type (left) and F19P (right) alloforms, and the data is summarized in Table S1.† The red circles on Fig. 1 represent the results of the calculated CCS and FRET-efficiencies as described above. It was not possible to obtain an experimental CCS value for the 3+ charge state since the ion count was extremely low under all electrospray conditions and thus it is the calculated CCS value that is plotted *in lieu* of an experimental value for both alloforms. There is a close agreement between the experimental and theoretical data points, particularly in the case of the 4+ and 5+ charge states of both alloforms. For the 3+ FRET efficiencies, since the calculated chromophore separation is typically <15 Å, it is impossible to eliminate a contribution from Dexter energy transfer or a breakdown in the Forster relation.^41^

From Fig. 1 it is possible to gain an overview into how the addition of protons changes the peptide structure. There is a large change in the FRET efficiency when the charge state is changed from 3+ to 4+. A large change in the calculated CCS is also observed, and although there is no experimental data for the 3+ charge state, the close agreement between theory and experiment for the other data points strengthens the case for this increase being real. Hence it can be intuited that there must be a correspondingly large change in the secondary structure in these two charge states. Contrastingly, only a small change in the CCS is observed for the 4+ and 5+ charge states, which suggests that the global structural motif of the peptide remains essentially unaltered. However, there is a larger relative decrease in the FRET efficiency between these two charge states. This is suggestive of an overall increase in the extension of the peptide, although for the F19P alloform the change in FRET efficiency from 4+ to 5+ is encompassed in the error bars for the 4+ FRET efficiency. This variance in the FRET-efficiency was measured consistently and appears to be a genuine uncertainty, perhaps indicating a greater flexibility for the relative position of the chromophores. Finally, there is a further large change in the CCS when comparing the 5+ and 6+ charge states, and a smaller decrease in the FRET efficiency. This suggests a further change in global structure, but one that would appear to leave the overall chromophore separation unaltered. However, it must be noted that as a FRET efficiency of 0 is approached, a relatively small change in FRET efficiency can correspond to a large change in the chromophore separation.

**Fig. 2:**
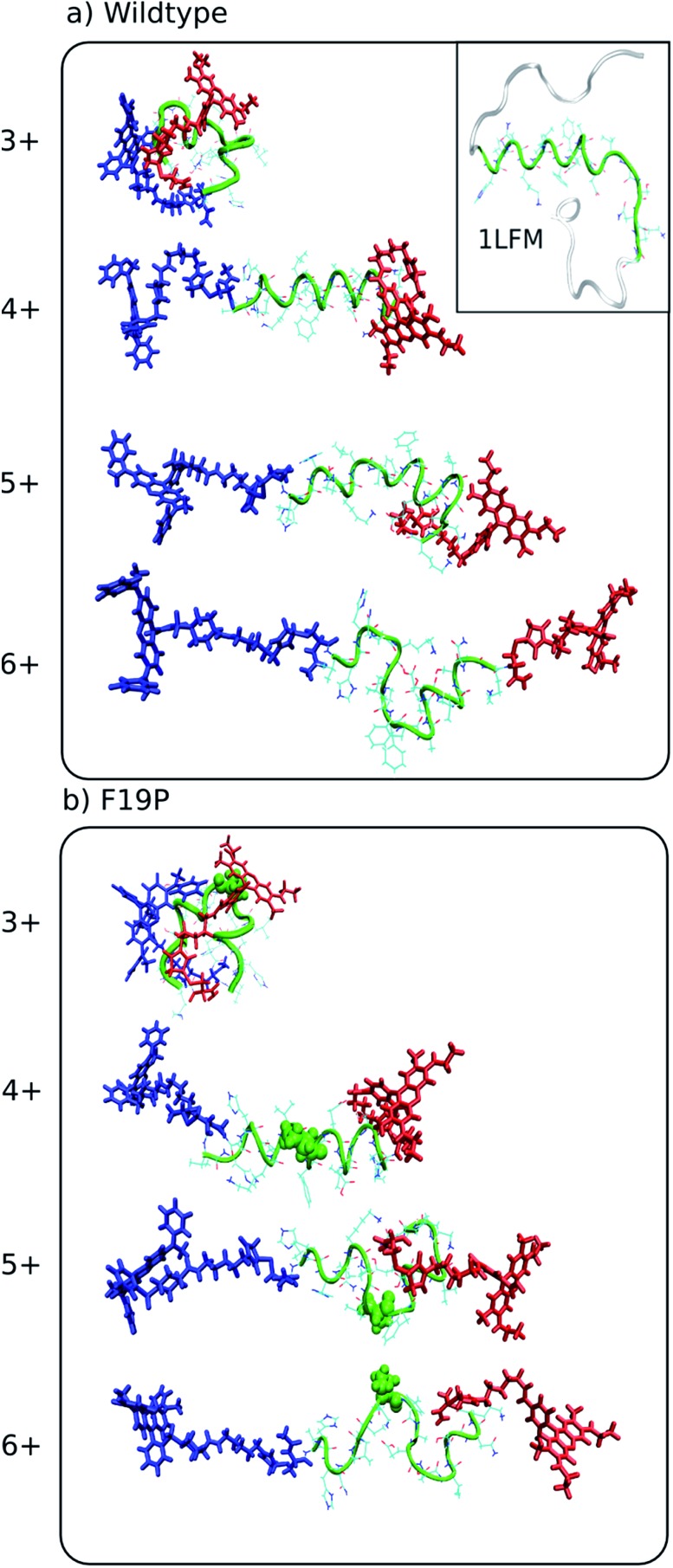
Representative structures simulated at 292 K of the dominant conformational family of the different charge states of the wild (top) and F19P (bottom) alloforms of Aβ_12–28_. Here, the donor chromophore (grafted to the C-terminal residue) is shown in red, the acceptor chromophore in blue, and the peptide backbone in green. The inset on the top panel shows the NMR structure (pdb database file ; 1LFM) for the full amyloid beta protein, with the 12–28 region highlighted in green. The corresponding figure of the ensembles with the alternative chromophore grafting location is shown in Fig. S2.†

**Fig. 3:**
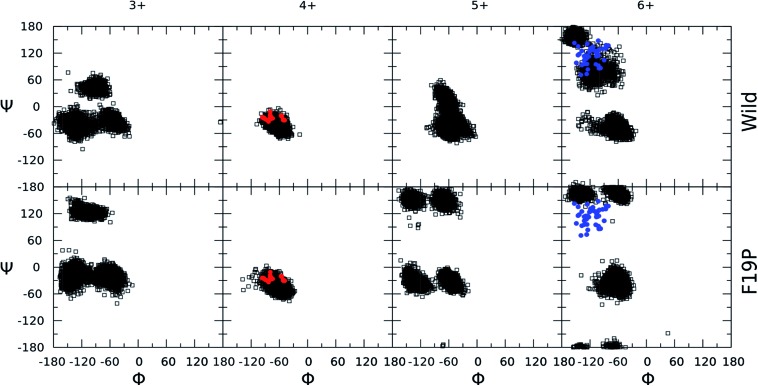
Ramachandran plots for the hydrophobic core region (residues 17–21) of the different charge states of the wild (top) and F19P (bottom) alloforms of Aβ_12–28_ (donor chromophore is grafted to the C-terminal residue). Black squares correspond to the 5 dihedral angle (*Φ*,*Ψ*) pairs of residues 17–21 for all the structures computed at 292 K. Red dots indicate the corresponding dihedral angles of the partially folded solution structure from the pdb file ; 1LFM. Blue dots indicate the dihedral angles of the A-chains in the pdb file ; 2BEG. The corresponding figure of the ensembles with the alternative chromophore grafting location is given in Fig. S3.†

Since the theoretical and experimental data is in excellent agreement, it is possible to examine representative structures for the charge states of each alloforms, shown in Fig. 2, to confirm that such trends as described in the previous paragraph are observed. Although such a visual comparison of representative structures can be illustrative, a more objective measure of structural differences is obtained by monitoring the changes in the dihedral angles of the peptide backbone – for amino acids in the hydrophobic core – in a Ramachandran plot, which is shown in Fig. 3 for the C-terminal donor chromophore and Fig. S3† for the N-terminal donor chromophore.

The first point that must be established is whether the presence of the chromophores is greatly perturbing the peptide secondary structure as compared to the un-grafted system. The molecular dynamics simulations reveal that – for a given alloform and charge state – the chromophores are very flexible due to the long C_5_ linker chain, whilst the peptide undergoes only small relative changes once a low energy structure is adopted. Additionally, the same trends in structural motif as a function of charge state are observed for both N- and C-terminally grafted QSY7, with only a greater degree of random-coil like nature for the C-terminally grafted F19P species observed. From this, the structural influence of the chromophores reduces to the existence of two positive charges at the N- and C-terminus.

A previous gas phase study of the structure of doubly and triply protonated Aβ_12–28_ found that there was a transition from globular to helical conformation with addition of a third proton to go from 2+ to 3+ charge state.^22^ A similar change in secondary structure is observed in the doubly grafted species in Fig. 2, although for singly (3+) and doubly (4+) protonated peptide. It thus appears that although similar structural trends are observed in these two systems, the presence of the N- and C-terminal charges of the chromophores is inducing the transition from coil-like to helical to occur at a lower peptide protonation state.

In order to recognize any commonalities between the physiologically relevant solution phase structure of the Aβ_1–42_ and the gas phase structure of the doubly grafted Aβ_12–28_ studied here, a visualization of the NMR structure of Aβ_1–42_ (pdb code ; 1LFM) is included as an inset to Fig. 2 with the 12–28 region highlighted in green. There is a clear helical motif for the 12–28 region, which is also observed for the 4+ charge state in both wild and F19P alloforms. That the 4+ charge state possesses a similar structural motif to the solution structure may be due to there being the same net charge on the peptide in both cases; the peptide being doubly protonated in doubly grafted Aβ_12–28_ and the 4 basic and 2 acidic amino acids in the 12–28 fragment, giving the net charge of 2+. To explore this similarity further, the backbone dihedral angles in the hydrophobic core region of Aβ_1–42_ are shown as red circles on 4+ Ramachandran plots of Fig. 3 (and Fig. S3†). An almost quantitative correspondence of the backbone conformation in the two systems is evident, providing striking evidence for the mimicking of native secondary structure in the doubly grafted species. The structural similarity of the 12–28 fragment may be attributed to the presence of the N- and C-terminal chromophores mimicking the presence of the missing N- and C-terminal peptide in Aβ_12–28_.

Contrastingly, the hydrophobic core region presents two alternative backbone configurations in the 3+ species which are completely absent from the solution phase structure. Indeed, the 3+ charge state presents a globular random-coil type structure in both alloforms. The large change in the structural motif – from globular random-coil to alpha-helical – is as predicted from the change in FRET efficiencies shown in Fig. 1.

In the 4+ charge state of the WT alloform, both lysine residues K16 and K28 carry the positive charges, and this additional Coulomb repulsion causes the observed unfolding. The F19P alloform behaves in a qualitatively similar way to the wild alloform for the 3+ and 4+ charge states, as evidenced by comparison of the hydrophobic core dihedral angle data of Fig. 3. In both cases there is a change in structure from globular random-coil of the 3+ charge state to extended helical in the 4+ charge state.

In the 5+ charge state, the additional proton must reside on one of the histidine residues, H13 or H14, since the N-terminus is acetylated. It is found that, for both possible grafted species, it is energetically favorable for the third proton to be located on the H14 residue. This may be due to the fact that the K16 and H14 are located on opposite sides of one of the helical turns, allowing the charges to maintain a maximum separation whilst retaining the favoured helical motif. Through the absence of further major charge-solvating effects in the gas-phase peptide with overall positively charged residues, the maximization of inter-charge separation as driving force for the observed structural changes upon protonation seem highly plausible.^43^,^44^

Protonation of the 4+ charge state to the 5+ charge state results in an overall elongation of both systems as evidenced by the decrease in FRET-efficiency and the small increase in CCS. There is a clear distortion of the helical motif in both alloforms, particularly in the hydrophobic core region. Fig. 3 shows a clear deviation from the “all-alpha” configuration of the 4+ charge state in both alloforms, but the nature of this deviation is strikingly different. In the WT alloform the deviation results in a distortion of the helical motif rather than a complete unfolding. The F19P alloform shows a more complete unfolding of the helical motif resulting in a significant random-coil character in the dihedral angle plot of Fig. 3. This emerging difference could indicate that the helix-stabilizing interactions cannot compensate for the charge-induced structural strain the F19P alloform, indicating a weakening of the stability of the helix as compared to the WT.

The addition of a fourth proton to the H13 residue to give a maximally charged 6+ species results in a complete disruption of the helical motif in the N-terminal region of both alloforms. Although similar behavior is observed for both alloforms in terms of this structural transition, there is a pronounced difference in the structural motifs presented by the hydrophobic core region. For the F19P alloform, the difference between 5+ and 6+ charge states lies mainly in further loss of helical motifs and a concurrent increase in random-coil character. The WT alloform also shows significant deviation from the helical structure, but a significant β character is now displayed.

**Scheme 1:**
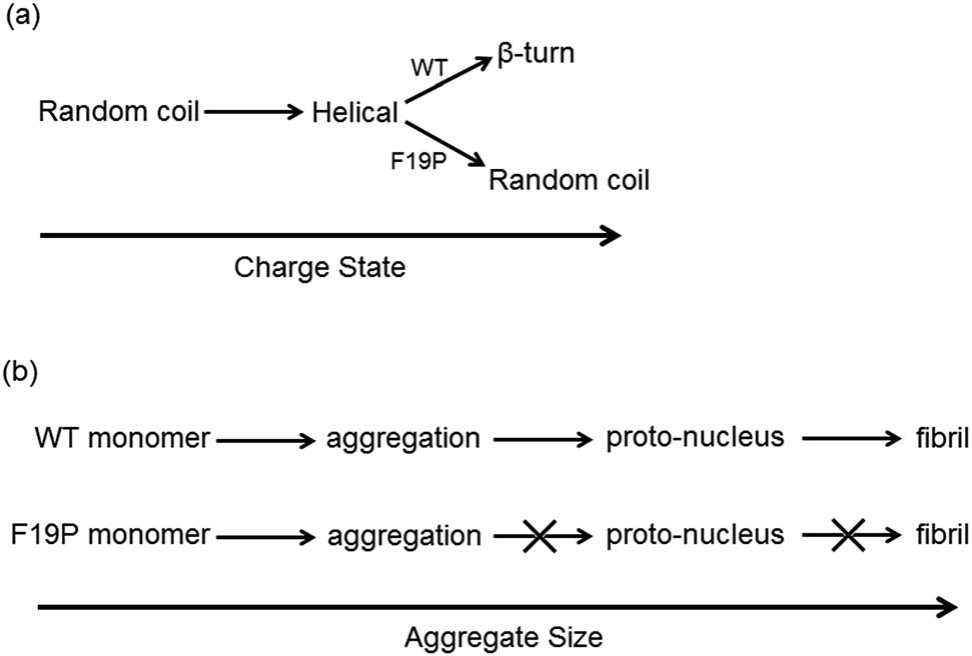
Summary of monomer structural transition with charge state (a) and proposed aggregation scheme (b) for the wild and F19P alloforms.

Since it is well established that large aggregates of Aβ are arranged as β-sheets, it has been suggested that the conformational change from helical to β-like structures in the monomer are an important step in the aggregation process.^45^,^46^ The dihedral angles for the hydrophobic core region of the Alzheimer's amyloid fibril (pdb structure ; 2BEG) are included in Fig. 3 for the 6+ charge states of the two alloforms as blue circles. The WT alloform possesses structures in which the same ranges of dihedral angles as present in the fibril are present, indicating that a fibril-like conformation is present in the WT. In contrast, there is no evidence of population of fibril-like monomer structures in the F19P alloform, which shows only random-coil motifs. The difference in the structural motifs as a function of charge state for the two alloforms is summarized in Scheme 1(a).

Both F19P and WT alloforms of the Aβ_1–42_ protein has been studied in the gas phase previously by Bernstein *et al.*, who concluded although both alloforms form small aggregates it is only the wild-type in which further oligomerization takes place to form large fibril-like structures.^12^ This observation lead Bernstein *et al.* to the conclusion that there is a key proto-nucleus which is vital for the formation of fibrils which are formed by aggregates of the WT – but not the F19P alloform, although the exact nature of this postulated proto-nucleus is unknown.^27^ One such model for understanding fibril self-assembly implies that at a certain aggregate size a transition between a random-coil and β conformation is observed, with the β-sheet fibril acting as a nucleus for further oligomerization.^46^ Bleiholder *et al.* found evidence for the existence of para-nuclei in the aggregation of important fragments of various naturally occurring fibril forming proteins, but not in the case of a peptide sequence which forms isotropic aggregates.^47^

The data presented here allows us to postulate a tentative hypothesis for the difference in aggregation behavior of the two alloforms based on the behavior of the hydrophobic core in the monomer discussed above and summarized in Scheme 1(a). The presence of the structural transition from the predominantly helical form of the 4+ and 5+ charge states to the significant proportion of β-turn-like structures in the 6+ for the WT alloform indicates that a structural transition similar to that suggested as resulting in the fibril proto-nucleus is possible, Scheme 1(b). Contrastingly, this structural transition is not observed in the F19P alloform. As such, the structural transition that is postulated to occur to form the proto-nucleus does not happen in the F19P alloform and thus the self-aggregation into extended fibril-like structures cannot occur. In this case, it could be expected that the isotropic aggregates observed by Bleiholder *et al.* observed in similar non-fibril forming species may form.

## 4 Conclusions and prospects

The structure of the different charge states of the 12–28 fragment of the amyloid beta protein have been studied by a combination of ion mobility mass spectrometry, action-FRET and REMD simulations. The addition of a second experimental dimension has allowed a thorough examination of the theoretical methods used here to reproduce the experimental values. In particular, the small changes in the hydrophobic core observed between the 4+ and 5+ charges states was illuminated by the second experimental dimension and allowed extraction of more information than either method alone could have. The presence of two experimental parameters further constrained the conformational landscape than can be probed by MD simulation and hence provided a higher degree of confidence that the calculated structures reproduce the experimentally observed conformation ensemble. Although the IMS and action-FRET experiments were performed separately on the systems reported in this work, the complementarity of the two methods in terms of the structural information accessible strongly suggests that a future coupling of the two measurements is a desirable union. This will be of particular importance when it comes to consideration of larger protein systems where there are multiple conformational families co-existing for a given charge state. In this case, it will be possible to perform conformationally resolved measurements in both IMS and action-FRET dimensions of an experiment, leading to a more robust characterization of, for example, the transition between folded and unfolded states of proteins as previously investigated by IMS alone.

For the amyloid beta fragment peptides, the influence of the charge state on the structure was studied. The charge state had a large influence on the structure, with a transition between random coil and helical being observed when going from singly to doubly protonated (3+ and 4+ species). Addition of a third proton on the peptide was found to destabilize the helix in the WT alloform and cause partial unfolding in the F19P alloform. The addition of a fourth proton to the peptide, giving the maximally charged 6+ species, caused unfolding of the helix in both alloforms.

Analysis of the dihedral angles of the peptide backbone in the hydrophobic core region of the two alloforms, showed that although the two alloforms exhibit a globally similar behavior as a function of charge state, there are significant differences in the structural motifs of the hydrophobic core. Comparison to the dihedral angles of the solution phase structure of both monomer and fibrillar Aβ_1–42_ showed that the helical to β-turn conformational transition that takes place during aggregation only occurs in the WT alloform, whilst the F19P alloform presents a random-coil structure. Hence, it was postulated the early propensity of the F19P alloform to undergo a helical to random-coil conformational transition prevents formation of the fibrillar proto-nucleus and thus the F19P alloform does not form extended fibrillar aggregates. The importance of such a transition in Aβ peptides potentially provides a glimpse into the aggregation propensity of other Aβ mutants. Ultimately, the possibility of a link between helix stability of the hydrophobic core and the propensity for aggregation may provide a novel target for potential drug design.

## Supplementary Material

Supplementary information
