# Correction: Conformational changes in amyloid-beta (12–28) alloforms studied using action-FRET, IMS and molecular dynamics simulations

**DOI:** 10.1039/c5sc90069g

**Published:** 2015-11-18

**Authors:** Steven Daly, Alexander Kulesza, Frederic Poussigue, Anne-Laure Simon, Chang Min Choi, Geoffrey Knight, Fabien Chirot, Luke MacAleese, Rodolphe Antoine, Philippe Dugourd

**Affiliations:** a Université de Lyon , F-69622 , Lyon , France . Email: philippe.dugourd@univ-lyon1.fr; b CNRS et Université Lyon 1 , UMR5306 , Institut Lumière Matière , France; c CNRS et Université Lyon 1 , UMR 5280 , Institut des Sciences Analytiques , France

## Abstract

Correction for ‘Conformational changes in amyloid-beta (12–28) alloforms studied using action-FRET, IMS and molecular dynamics simulations’ by Steven Daly *et al.*, *Chem. Sci.*, 2015, **6**, 5040–5047.



## 

There has been a consistent error in the name of the PDB code which was provided in the paper. The code given was 1LFM, but should have read ; 2LFM. There are 3 instances of this in the manuscript and one in the ESI.

(1) The inset of Fig. 2 and the figure caption should appear as:

**Fig. 2:**
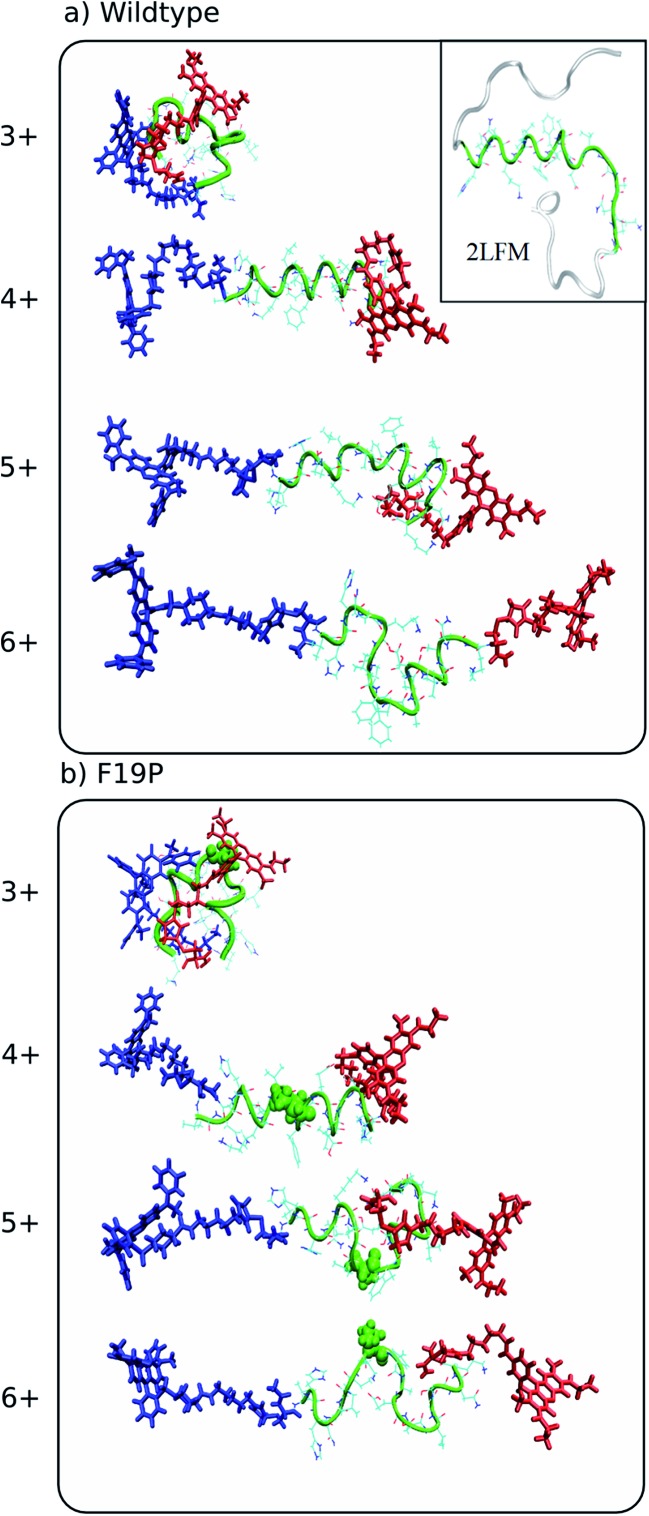
Representative structures simulated at 292 K of the dominant conformational family of the different charge states of the wild (top) and F19P (bottom) alloforms of Aβ_12–28_. Here, the donor chromophore (grafted to the C-terminal residue) is shown in red, the acceptor chromophore in blue, and the peptide backbone in green. The inset on the top panel shows the NMR structure (pdb database file ; 2LFM) for the full amyloid beta protein, with the 12–28 region highlighted in green. The corresponding figure of the ensembles with the alternative chromophore grafting location is shown in Fig. S2.

(2) On page 5044 (manuscript page 5), right hand column, line 12. The PDB code should read 2LFM rather than ; 1LFM.

(3) On page 5044, in the caption of Fig. 3. The caption should read:

Ramachandran plots for the hydrophobic core region (residues 17–21) of the different charge states of the wild (top) and F19P (bottom) alloforms of Aβ_12–28_ (donor chromophore is grafted to the C-terminal residue). Black squares correspond to the 5 dihedral angle (*Φ*,*Ψ*) pairs of residues 17–21 for all the structures computed at 292 K. Red dots indicate the corresponding dihedral angles of the partially folded solution structure from the pdb file ; 2LFM. Blue dots indicate the dihedral angles of the A-chains in the pdb file ; 2BEG. The corresponding figure of the ensembles with the alternative chromophore grafting location is given in Fig. S3.†

(4) In the ESI, the caption of Fig. S3 should read:

Fig. S3. Ramachandran plots for the hydrophobic core region (residues 17–21) of the different charge states of the wild (top) and F19P (bottom) alloforms of Aβ_12–28_ (donor chromophore is grafted to the N-terminal residue). Black squares correspond to the 5 dihedral angle (*Φ*,*Ψ*) pairs of residues 17–21 for all the structures computed at 292 K. Red dots indicate the corresponding dihedral angles of the partially folded solution structure from the pdb file ; 2LFM. Blue dots indicate the dihedral angles of the A-chains in the pdb file ; 2BEG.

The Royal Society of Chemistry apologises for these errors and any consequent inconvenience to authors and readers.

