# Supplementary material for: Conformational changes in amyloid-beta (12–28) alloforms studied using action-FRET, IMS and molecular dynamics simulations
Source: Chem Sci. 2015 Jun 18;6(8):5040–7. doi: 10.1039/c5sc01463h (PMC6088554; doi:10.1039/c5sc01463h)
Supplement: Supplementary file 1 [file SC-006-C5SC01463H-s001.pdf]

Electronic Supplementary Material (ESI) for Chemical Science.
This journal is © The Royal Society of Chemistry 2015

Conformational changes in Amyloid-Beta (12-28) alloforms studied using action-FRET, IMS and molecular dynamics simulations.

Steven Daly[a,b], Alexander Kulesza[a,b], Frederic Poussigue[a,c], Anne-Laure Simon[a,b], Chang Min Choi[a,b], Geoffrey Knight[a,b], Fabien Chirot[a,c], Luke MacAleese[a,b], Rodolphe Antoine[a,b], Philippe Dugourd[a,b]*.

[a] Université de Lyon, F-69622, Lyon, France.

[b] CNRS et Université Lyon 1, UMR5306, Institut Lumière Matière.

[c] CNRS et Université Lyon 1 UMR 5280, Institut des Sciences Analytiques

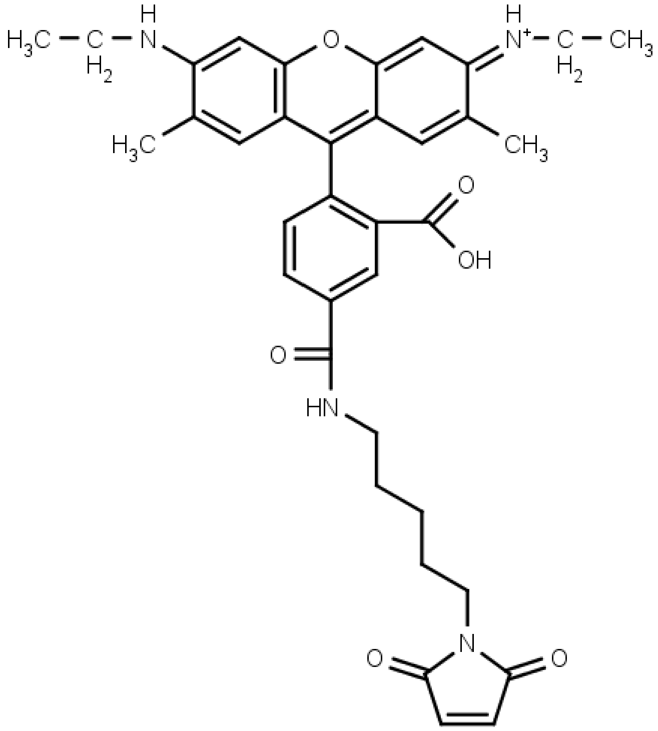

R575

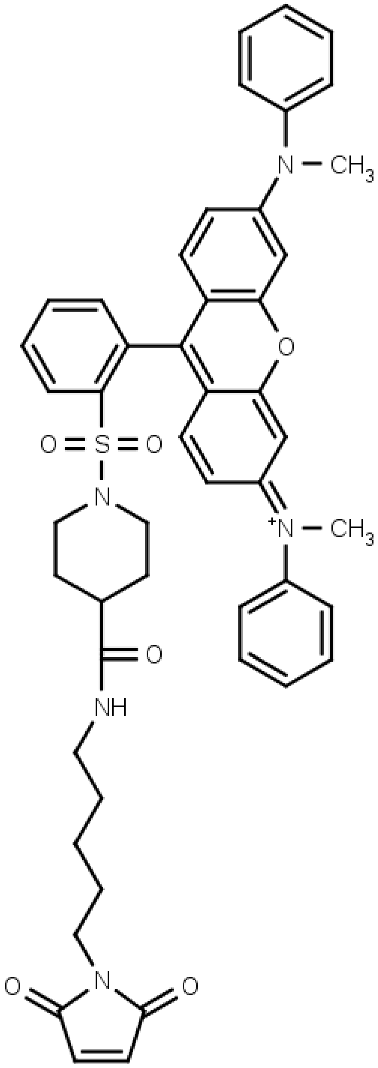

QSY 7

ace-CVHHQKLVFFAEDVGSNKC-NH_2 - wild

ace-CVHHQKLVPFAEDVGSNKC-NH_2 - F19P

Figure S1. The structures of carboxyrhodamine 575 C_5 maleimide (donor chromophore, top left), QSY 7 C_5-maleimide (acceptor chromophore, top right), and the two alloforms of the $A\beta_{12\text{-}28}$ fragment, which differ by a mutation of phenylalanine to proline at position 19 (red) and both possess two terminal cysteines to allow grafting of the chromophores (green).

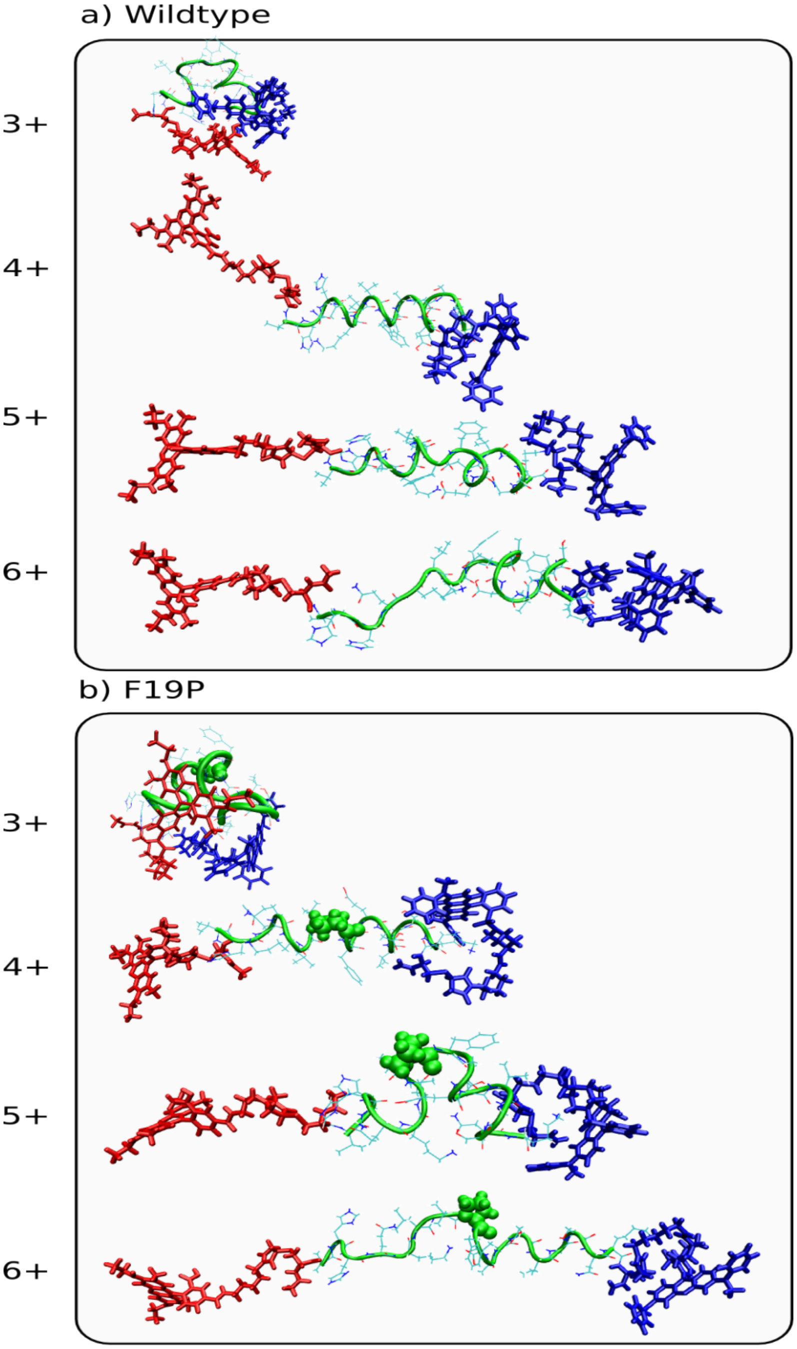

Figure S2. Representative structures simulated at 292K of the dominant conformational family of the different charge states of the wild (top) and F19P (bottom) alloforms of A$\beta_{12\text{-}28}$. Here, the donor chromophore (grafted to the N-terminal residue) is shown in red, the acceptor chromophore in blue, and the peptide backbone in green. The same trends in secondary structure as a function of charge state are observed for both of the possible doubly-grafted species of both alloforms, showing that the grafting location of the chromophore has only minor influence on the peptide structure.

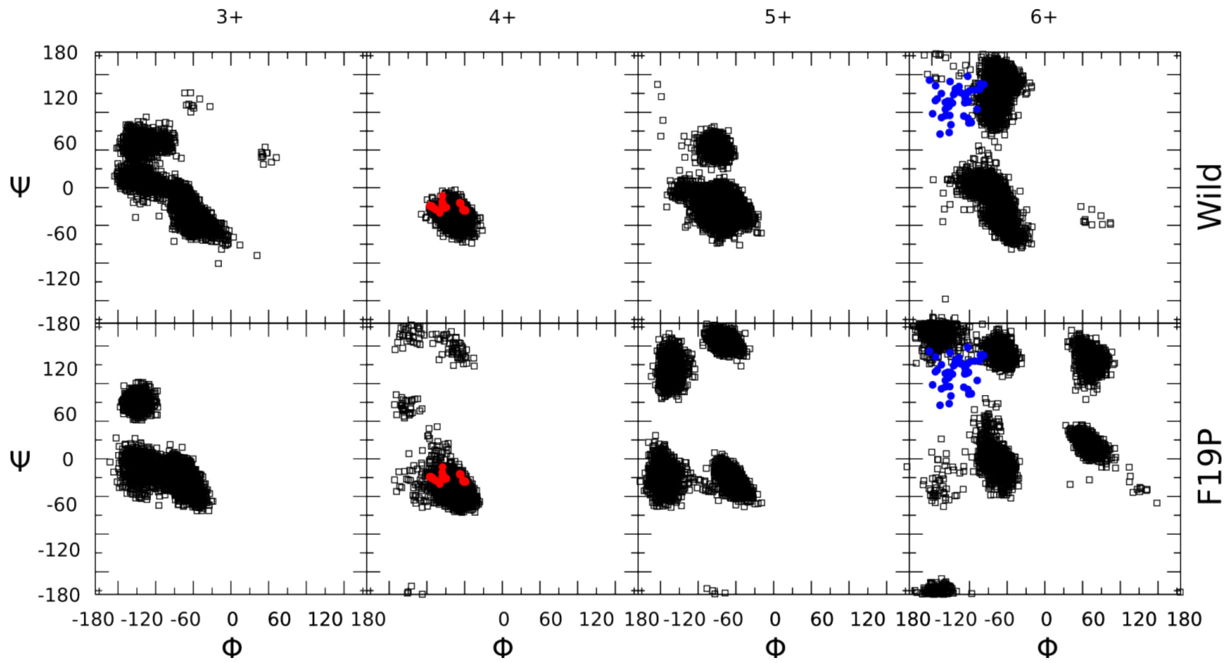

Figure S3. Ramachandran plots for the hydrophobic core region (residues 17-21) of the different charge states of the wild (top) and F19P (bottom) alloforms of A$\beta_{12\text{-}28}$ (donor chromophore is grafted to the N-terminal residue). Black squares correspond to the 5 dihedral angle (Φ,Ψ) pairs of residues 17 – 21 for all the structures computed at 292 K. Red dots indicate the corresponding dihedral angles of the partially folded solution structure from the pdb file 1LFM. Blue dots indicate the dihedral angles of the A-chains in the pdb file 2BEG.

Charge	WT				F19P			
	Exp. FRET eff.	Calc. FRET eff.	Exp. CCS	Calc. CCS	Exp. FRET eff.	Calc. FRET eff.	Exp. CCS	Calc. CCS
3+	1.11 ± 0.06	0.97	n/a	611	0.85 ± 0.06	0.97	n/a	575
4+	0.31 ± 0.06	0.29	801 ± 24	805	0.22 ± 0.15	0.30	784 ± 24	790
5+	0.16 ± 0.05	0.17	826 ± 25	825	0.13 ± 0.05	0.15	807 ± 25	804
6+	0.10 ± 0.05	0.063	963 ± 50	915	0.10 ± 0.03	0.049	921 ± 25	903

Table S1. Summary of the experimental and calculated collision cross section and action-FRET data presented in figure 1.
